# Human acireductone dioxygenase (HsARD), cancer and human health: Black hat, white hat or gray?

**DOI:** 10.3390/inorganics7080101

**Published:** 2019-08-18

**Authors:** Xinyue Liu, Thomas C. Pochapsky

**Affiliations:** 1 Department of Chemistry, Brandeis University, Waltham, Massachusetts 02454, United States; 2 Department of Biochemistry, Brandeis University, Waltham, Massachusetts 02454, United States

**Keywords:** *AD11*, nickel-dependent enzyme, methionine salvage pathway, methionine, S-adenosylmethionine (SAM), methylthioadenosine (MTA), enolase phosphatase 1 (ENOPH1), polyamine, matrix metalloproteinase MT1 (MT1-MMP)

## Abstract

Multiple factors involving the methionine salvage pathway (MSP) and polyamine biosynthesis have been found to be involved in cancer cell proliferation, migration, invasion and metastasis. This review summarizes the relationships of the MSP enzyme acireductone dioxygenase (ARD), the *ADI1* gene encoding ARD and other gene products (ADI1GP) with carcinomas and carcinogenesis. ARD exhibits structural and functional differences depending upon the metal bound in the active site. In the penultimate step of the MSP, the Fe^2+^ bound form of ARD catalyzes the on-pathway oxidation of acireductone leading to methionine, whereas Ni^2+^ bound ARD catalyzes an off-pathway reaction producing methylthiopropionate and carbon monoxide, a biological signaling molecule and anti-apoptotic. The relationship between ADI1GP, MSP and polyamine synthesis are discussed, along with possible role(s) of metal in modulating the cellular behavior of ADI1GP and its interactions with other cellular components.

## 1. Introduction

**Figure 1.**
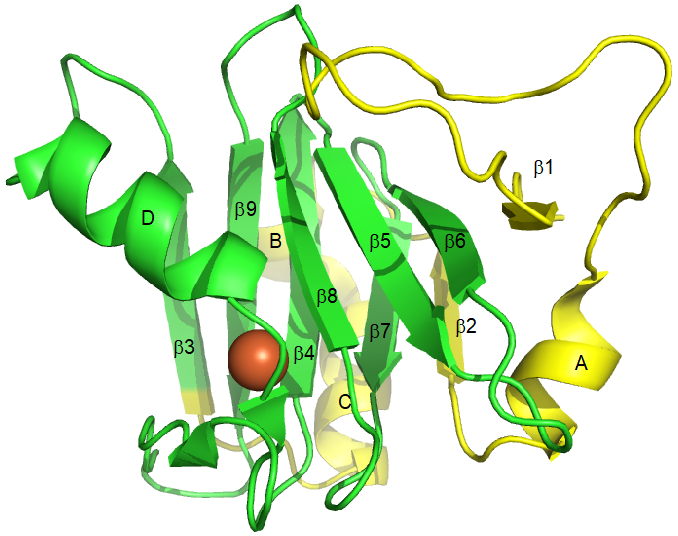
Crystal structure 4QGN [2] of HsARD (ADI1GP), showing arrangements of secondary structures referred to in the text. Residues in yellow are truncated in SipL (*s*ubmergence-*i*nduced-*p*rotein-*l*ike). Bound metal is shown as an orange sphere. Secondary structural features correspond to residue numbers as follows: β1, A4-Y6; helix A, G27-R33; β2, L37-K40; helix B, K45-D49; helix C, P50-R59; β3, W63-I69; helix D, N76-E86; β4, E94-G101; β5, G103-R108; β6, W114-M119; β7, D123-L127; β8, H133-V137; β9, T143-F149. C-terminal residues 163-178 are not shown; while helical in the crystal they are disordered in solution (T. Pochapsky and X. Liu, unpublished results).

**Figure 2.**
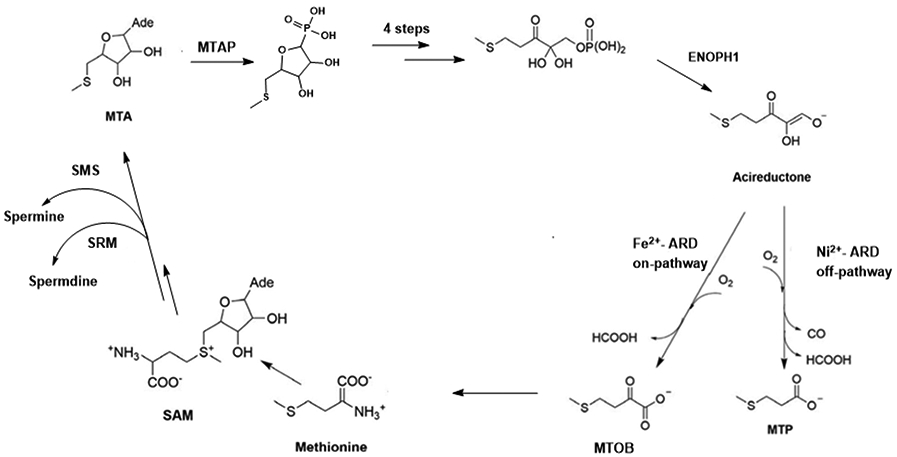
The methionine salvage pathway. .

Acireductone dioxygenase (ARD) is a metalloenzyme of the cupin superfamily that is ubiquitous among aerobic cellular organisms. It has been identified in bacteria, plants, fungi and animals. Those ARDs that have been structurally characterized all exhibit the standard cupin fold, a double-stranded β-helix domain fringed by three pseudosymmetrically arranged α-helices (Figure 1). In all organisms in where it has been characterized, ARD functions primarily in the methionine salvage pathway (MSP). As the name implies, ARD catalyzes the oxidative cleavage of the penultimate intermediate in the pathway (1-(thiomethyl)-3-keto-4,5-dihydroxy-pent-4-ene, acireductone in Figure 2) to formate and the ketoacid precursor of methionine, MTOB (4-methylthio-2-oxobutyrate).

ARD was first identified in the methionine salvage pathway of the bacterium *Klebsiella*, as part of an effort to better understand mechanisms of methionine metabolism.[1] It had been known for some time that, unlike the cell lines from which they are derived, many carcinomas are either strictly or partially dependent on an external supply of methionine (Met) for survival (Table 1). However, it was (and is yet) unclear whether this is due to an inability to recover Met from S-adenosylmethionine (SAM or AdoMet) or simply Met usage in excess of what normal cellular processes require that cause this dependence. The efforts of the Abeles group in the late 1980s and 1990s were aimed at a clearer understanding of the methionine recycle and salvage pathways. While methionine recycling involves S-methylation of homocysteine (a by-product of activated methyl transfers from SAM), methionine salvage retains only the S-CH_3_ group of the original methionine, with all of the other carbon atoms of methionine originating from the ribose moiety of methylthioadenosine (MTA). MTA is produced as the result of ethylene biosynthesis in plants and polyamines in other organisms (Figure 2).

A remarkable discovery by that group was that the enzyme eventually named ARD could be isolated from *Klebsiella pneumoniae* (later reclassified as *K. oxytoca)* in two chromatographically separable forms with different enzymatic activities. While the polypeptides of both isoforms were identical, it was found that ARD to which Fe^2+^ was bound catalyzed the on-MSP reaction resulting in methionine biosynthesis, but if Ni^2+^ was bound, an off-pathway reaction occurs, leading to the formation of formate, methylthiopropionate and carbon monoxide (CO).[1, 3] Subsequent research showed that the same four residues bound the metal in either case, and the structural differences between the two enzymes are the result of subtle differences in metal-ligand bond lengths, which trigger the formation of secondary structural features in the Ni-bound form that are absent in Fe-ARD.[4] The metal-dependent dual functionality of ARD has been confirmed in vitro for mammalian ARDs from mouse (*Mm*ARD) and humans (*Hs*ARD).[5, 6]

The metal binding motif of ARD is 3-His 1-Glu, unlike the 2-His 1-Glu/Asp scheme used by α-ketoglutarate (KG)-dependent dioxygenases (also members of the cupin superfamily). The 2-His 1-Glu/Asp ligands in the KG-dependent dioxygenases are located within the wide end of the cupin β-barrel, where they are relatively immobilized. The extra histidine ligand in ARD is located in a turn on the edge of the barrel, adjacent to a loop that appears to regulate metal-dependent conformational differences between the Fe- and Ni-bound enzymes via changes in hydrogen bonding patterns.[4] While the conformational differences between FeARD and NiARD have only been confirmed for the *Klebsiella* enzyme, the fact that the *Mm*ARD and *Hs*ARD enzymes are also separable chromatographically in their Fe- and Ni-bound forms, as well as their differential thermal stabilities, strongly support the likelihood of a metal-dependent conformational shift in these enzymes as well. Whether the conformational shift is responsible for or merely coincident with the different activities of the ARD isozymes is not known. These and related questions have been thoroughly reviewed recently, and interested readers are referred to that review.[7] Rather, we will look at the intriguing and (often) confusing links between the ARD gene product and human health, in particular, cancer and carcinogenesis.

## 2. *ADI1* and ADI1GP

**Figure 3.**
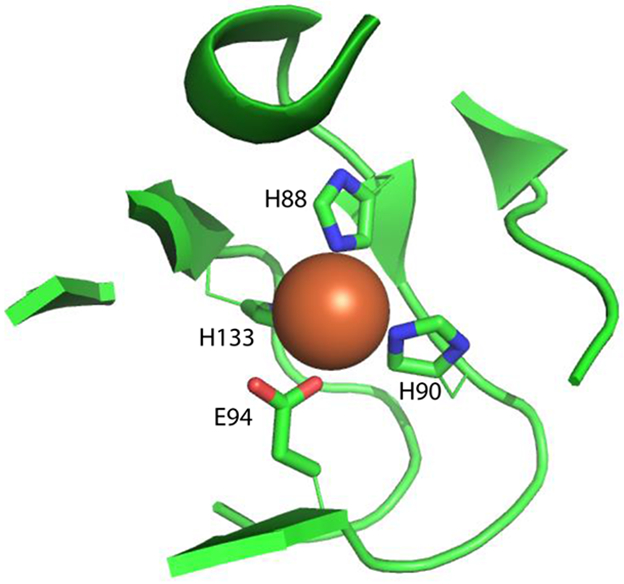
Metal ligation scheme in *Hs*ARD (PDB entry 4QGN). Protein-based ligands are His88, His90, Glu94 and His133. The metal is reported to be Fe^3+^, although how this was determined is not reported.

*Hs*ARD is encoded by the *ADI1* gene, which is located on chromosome 2 at locus 2p25.3 and is comprised of four exons. Given that different forms of *ADI1* gene product may have different enzymatic and regulatory effects in vivo, we will refer to them collectively as ADI1GP. The full length ADI1GP is 179 amino acids, with metal ligands provided by His 88, His 90, Glu 94 and His 133 (Figure 3). A partial ADI1GP, discovered prior to identification of ARD, was termed SipL (*s*ubmergence-*i*nduced-*p*rotein-*l*ike) based on homology with a submergence-induced rice plant gene (*Sip*) that was subsequently shown to be an ARD (OsARD). [6, 16-18] SipL is a truncated ADI1GP, missing 63 residues from the N-terminus. Given that residue 64 of the ADI1GP is a Met, and a portion of the second exon is missing in the SipL sequence, it is likely that SipL is the result of a truncated ribosomal translation rather than an alternate mRNA splicing. The deletion removes strands β1 and β2 from the β-helix, as well as helix A (see Figure 1) but retains all four metal binding ligands. Both SipL and the full-length ADI1GP were found to be involved in human hepatitis C virus replication, permitting viral infection of otherwise resistant tissues.[19-21]

Multiple tissues show high levels of ADI1GP based on Western blot analysis, including liver, kidney, prostate, thyroid, and skeletal muscle. Lower expression levels are seen in the adrenal gland, trachea, spinal cord and stomach. Only trace amounts of *ADI1* mRNA have been observed in heart, brain, mammary gland, lymph node, bone marrow, placenta, bladder and leukocytes.[22] ADI1GP can be localized both in the cytosolic and nuclear compartments.[23, 24]

### 2.1. A role for ADI1GP in hepatitis C virus (HCV) infection

The first hints that ADI1GP might play important roles in disease progression were found even before the discovery of the enzyme, in the work by Yeh et al. on the role of SipL in hepatitis C infection (vide supra). The hepatitis C virus (HCV) is a blood-borne (+)-single-stranded RNA virus with a genome 9.6 kb in length. HCV is the major cause of chronic hepatitis worldwide with severe complications, including cirrhosis, hepatic failure and hepatocellular carcinoma. HCV entry into the cell begins with the HCV E2 coat protein specifically binding to four transmembrane domains of tetraspanin CD81 located on the hepatocyte surface. However, this interaction alone is not sufficient to cause infection, other cofactors appear to be required.[25, 26] Yeh et al. injected mouse hepatoma cells, Hepa1-6-CD81-SipL infected with HCV-positive serum into a mouse model, and detected HCV RNA by days 2-6 via RT-PCR. Immunofluorescence assays of resected tumor tissue identified SipL as a hepatic factor supporting HCV infection and replication, in combination with CD81 and other cofactors in an otherwise non-permissive cell line.[19] Further studies on mouse hepatomas verified that co-expressed human CD81 and SipL in Hepa1-6cells are permissive for HCV infection and replication. As noted above, SipL was identified to be a truncated version of ADI1GP. The group suggested the function of SipL in HCV infection was to bind with membrane-type 1 matrix metalloproteinase (MT1-MMP), thereby facilitating cell entry of HCV, albeit with low efficiency. [20] That group later showed that expression of ADI1GP alone can lead to a small amount of cell entry and replication of HCV, although the efficiency of cell entry and replication was enhanced significantly by co-expression with CD81. In addition, their replicon transfection experiments indicated ADI1GP expression did not increase replication efficiency. Hence, the group proposed expression of human ADI1GP could increase cell uptake of HCV, but not replication. [21]

A recent study indicated that an interaction between MT1-MMP (matrix metalloproteinase MT1) and ADI1GP was correlated with *lower* HCV RNA levels in Huh7.5 cells, with the MT1-MMP-ADI1GP interaction decreasing HCV cell entry. However, when ADI1GP was overexpressed, the inhibitory effect was reversed. These researchers proposed that interaction between ADI1GP and MT1-MMP would draw MT1-MMP away from interacting with CD81 conducive to HCV entry, thereby reversing the inhibitory effect.[23]

### 2.2. ADI1 in hepatocellular carcinoma (HCC)

Given the links between HCV infection and liver cancer, it would be reasonable to suspect that *ADI1* gene expression might be correlated with liver neoplasms. A recent paper by Chu et. al. found a negative correlation between ADI1GP expression and hepatocellular carcinoma (HCC) cell proliferation.[24] Western blot results indicated a significant reduction of ADI1GP in tumor tissues versus normal liver tissue in a group of 161 patients. The group performed short hairpin *ADI1*-mediated knockdown in human hepatocellular carcinoma cell lines J7 and Huh7, showing that depletion of ADI1GP markedly enhanced cell proliferation. On the other hand, overexpression of ADI1GP resulted in decreased cell proliferation. Terminal deoxynucleotidyl transferase dUTP nick-end labeling (TUNEL) assays indicated that AD1GP overexpression lead to a large increase in the rate of apoptosis. An mRNA analysis of the human hepatocellular carcinoma GSE14520 dataset from the NCBI Gene Expression Omnibus and the human hepatocellular carcinoma dataset from the Cancer Genome Atlas program showed *ADI1* mRNA level reductions in cancerous tissue with more substantial down-regulation of *ADI1* in later stages of HCC progression, a correlation supported by immunohistochemistry analysis.[24]

This group also claimed evidence that metal binding modulates ADI1GP effects on cell proliferation.[24] Over-expression of the ADI1GP mutant E94A in J7 and Huh7 cells showed a significant decrease of cell proliferation, while the H133A mutation did not change cell growth rates relative to untransformed cells, with a similar result observed on HCC tissue xenografts. While the authors suggested that these results implied a role for the on-pathway MSP function of *Hs*ARD in HCC growth repression, it is not clear why the removal of one ligand (E94) should be different from another (H133). Mutations of any of the four ligands in *Klebsiella oxytoca* ARD resulted in complete loss of metal binding and enzymatic function.[27]

The same group investigated altered gene expression levels due to ADI1GP overexpression. They found that caveolin-1 (CAV1) was consistently down-regulated both at the protein (Western blot) and mRNA levels, with the same effect observed in both tumor and normal tissues. Caveolins are a class of oligomeric proteins involved in caveolae formation.[28] CAV1 is involved in lipid transport, membrane trafficking and signal transduction.[29] Interestingly, CAV1 has also been implicated in oncogenic cell transformation, tumorigenesis, and metastasis.[28, 30] Additional mutation studies suggested that only the functional on-MSP ADI1GP would significantly down-regulate CAV1 expression, with CAV1 a downstream effector in ADI1-mediated repression. Due to the strong positive correlation between ADI1GP expression levels and SAM concentrations in the cell, the relation between CAV1 protein level and SAM levels were tested. The group found CAV1 expression decreases as SAM levels increase, with similar amounts of ADI1GP present, suggesting that ADI1GP inhibits *CAV1* expression via the MSP. Computational analysis of the NCBI database suggest ADI1GP levels to be negatively correlated with CAV1 levels in HCC patients.[24]

Given that SAM plays an important role in cellular methylation, it was proposed that increased levels of ADI1GP would increase SAM concentrations and result in *CAV1* gene methylation to suppress transcription. They proposed a regulatory mechanism where in non-cancerous HCC cells, high ADI1GP levels would generate a large amount of SAM, modulating genome-wide methylation (with 15% in gene promoter regions), resulting in tumor suppression. However, in cancerous hepatocytes, with less ADI1GP and less SAM available, alterations of genome methylation patterns might promote cell proliferation. While an intriguing possibility, the study does not exclude the potential participation of other factors in determining gene methylation patterns.[24]

### 2.3. ADI1GP induces apoptosis in prostate cancer cell lines

Further evidence that ADI1GP may be important in preventing or regulating carcinogenesis is that elevated *ADI1* expression level led to an increase in apoptosis in prostate cancer cell lines. Oram et al. observed down-regulation of *ADI1* in high-grade prostate tumor cell lines. Epithelial and stromal cells are the two major components of prostate. Non-cancerous prostatic hyperplasia tissue showed ADI1 mRNA expression in epithelial cells and little or no expression in stromal cells. Gleason grade 3 prostate tumor tissues had less ADI1GP than the benign specimen. A previous study using rat prostate epithelial cells and LNCaP epithelial cell lines expressed the ADI1GP ortholog ALP1.[22, 31]

By introducing the synthetic androgen miboleron (Mib) in epithelial LNCaP Cells and inhibitor CHX, the researchers has demonstrated that *ADI1* mRNA expression was regulated by Mib, and proposed that Mib directly induced *ADI1* expression. Androgen has previously found to be regulating ortholog *APL1* mRNA levels in rat. [22, 31]

In order to examine how metal binding to ADI1GP influences apoptosis and growth inhibition of the prostate cell lines, mutations of conserved metal-ligating residues were made. The group examined the localization of ADI1GP mutants in LNCaP and PC3 cells using fluorescence microscopy. Since no ADI1GP expression in stromal cell lines would be observed, the group studied PC3 to illustrate the apoptosis-promoting function of ADI1GP. For all the mutations, ranging from single to quadruple, on ADI1GP, PC3 cell lines all showed approximately the same apoptosis rates as wild type ADI1GP. In addition, the same ADI1GP mutants and WT in LNCaP cell lines resulted in decreased colony formation relative to controls, consistent with the results for LNCaP cells in their mRNA and tissue studies. In summary, these results suggest that the apoptosis-inducing effects of ADI1GP on stromal cells appear to be independent of metal binding.

On the other hand, apoptosis could be induced in pancreatic carcinoma, breast tumor, and HCC cell lines by supplementing growth media with the on-pathway MSP product of ADI1GP oxidation, MTOB.[32-34] Furthermore, other upstream metabolites (e.g. MTA, SAM) also induce apoptosis.[24, 32, 34, 35] Ornithine decarboxylase 1 (ODC), an enzyme from the polyamine biosynthesis pathway, also inhibits tumor growth.[22, 36] As such, further investigations are needed to confirm that enhanced apoptosis and tumor inhibition with elevated ADI1GP expression levels are truly independent of metal binding.

### 2.4. ADI1GP regulation of membrane-type 1 matrix metalloproteinase (MT1-MMP)

Perhaps the most intriguing and direct link between ADI1GP and cancer is the observation that ADI1GP appears to suppress the metastasis-promoting activity of MT1-MMP. ^[37],[31, 38]^ MT1-MMP, also called MMP-14, functions in the pericellular space on the cell surface, where it is involved in the degradation of extracellular matrix involved in cellular functions such as migration, proliferation, and the regulation of cell morphology.[26, 39] Due to its high expression level in cancerous tissue, MT1-MMP is believed to have a significant role in tumor metastasis via degradation of the extracellular matrix, freeing tumor cells to migrate. MT1-MMP activity is regulated by a transmembrane tail projecting into the cytoplasm. [26, 39, 40] Uekita and coworkers reported that ADI1GP down-regulates cell migration and invasion promoted by MT1-MMP. By comparing the interaction of ADI1GP with a FLAG-tagged MT1-MMP against that with an MT1-MMP mutant lacking the regulatory cytoplasmic tail, these researchers found that wild-type MT1-MMP formed a complex with the ADI1GP (which they called MTCBP-1) at the cytoplasmic tail of MT1-MMP. Further investigation showed ADI1GP to co-localize with MT1-MMP at the plasma membrane, and that ADI1GP was only recruited into the membrane fraction in the presence of the cytoplasmic tail. They showed ADI1GP co-expression specifically inhibited MT1-MMP-promoted cell migration but not other types of migration. That group also found that ADI1GP significantly reduces tissue invasion caused by MT1-MMP. They concluded that ADI1GP binding to the cytoplasmic tail of MT1-MMP regulates the activity of the enzyme towards the intercellular matrix, and reduced ADI1GP levels in tumor cell lines compared with the non-transformed fibroblasts would provide an advantage to the tumor cells for migration and proliferation.[17, 37, 41]

### 2.5. ADI1GP/MT1-MMP interaction restricts metastasis of pancreatic ductal adenocarcinoma (PDAC)

ADI1GP has also been found to restrict tumor metastasis by disrupting the interactions between MT1-MMP and F-actin in pancreatic ductal adenocarcinoma (PDAC). [42] Metastatic PDAC tumors are extremely aggressive and invasive, actively remodeling the actin-rich invadopodia that protrude into the extracellular matrix, facilitating invasion of nearby tissues.[43-45] Qiang et. al. suggested that ADI1GP may serve as an endogenous antimetastatic factor in PDAC. They showed that decreasing ADI1GP enhances invasive migration and increases the rate of extracellular matrix degradation in PDAC cell lines DanG, BxPC3, and Panc-1. Conversely, ADI1GP overexpression slows invasion, suppresses extracellular matrix degradation and reduces invadopodia counts in PDAC cells. Using fluorescence microscopy with WT and mutant MT1-MMP lacking the cytoplasmic tail, they showed that ADI1GP binds the cytoplasmic tail of MT1-MMP directly, inhibiting the invasive properties of PDAC. In addition, they found ADI1GP in the invadopodia disrupted the interactions between MT1-MMP and F-actin. ADI1GP was thus shown to be an intrinsic inhibitor of stromal remodeling through a direct interaction with MT1-MMP. The localization of ADI1GP to invadopodia significantly reduced the capacity of PDAC cells to invade and metastasize into peripheral tissues. [42]

## 3. Polyamines, MSP and cancer

Elevated polyamine (spermidine, spermine and putrescine) levels have long been known to be associated with tumors and tumorogenesis.[46] Polyamine biosynthesis and concentrations are tightly regulated under normal conditions, given their relationship with cell replication and proliferation. Increased polyamine levels are associated with malignant transformation, increased cell proliferation and preservation of neoplastic phenotypes.[46-49]

The polyamine precursor S-adenosylmethionine (SAM) plays multiple important roles in the cell. SAM is the principle methyl donor required for methylation of nucleic acids, phospholipids, histones, biogenic amines, and proteins.[50, 51] Low SAM levels are associated with chronic liver diseases.[52-54] Hepatocellular SAM concentrations were found to affect oxidative stress, mitochondrial function, hepatocellular apoptosis as well as malignant transformation.[50] Gene methylation has been proposed for tumor suppression, suggesting that SAM might be used for tumor suppression through its methylation function.[24] The processing of SAM metabolites depends upon its use. SAM-supported methylation reactions result in homocysteine production via the transsulfuration pathway, which produces S-adenosylhomocystine, followed by hydrolysis to form homocysteine, which then progresses via intermediate cystathionine to cysteine.[55]

The biosynthesis of polyamines makes use of the amino acid substituent on decarboxyated SAM, with spermidine synthase (SRM) yielding spermidine and spermine synthase (SMS) producing spermine. In either case, methylthioadenosine (MTA), the first committed intermediate of the MSP, is a byproduct (Figure 2). MTA has been shown to regulate gene expression, inhibit protein methylation, prevent cell proliferation, and regulate apoptosis. MTA also had a role in tumor development, cancer cell invasion and lymphocyte activation.[35] As such, MTA levels are also tightly regulated under normal conditions, regulation that depends heavily on a functional MSP, in which MTA is cleaved and phosphorylated to form 5’-methylthioribose-1-phosphate (MTR-1-P) catalyzed by 5’-methythioadenosine phosphorylase (MTAP). In normal cells, MTA is rapidly metabolized by MTAP. Studies have shown MTAP expression induces a significant reduction in intracellular polyamine levels as well as putrescine to total polyamine ratio changes.[56] Many malignant cell lines lack MTAP activity, and MTAP-deficient cells were found to secrete MTA.[35, 57, 58] In addition to SRM and SMS, ornithine decarboxylase 1 (ODC) also serve a key role in polyamines biosynthesis reacting with ornithine to produce putrescine. MTA inhibits SRM, ODC and strongly inhibits SMS.[35, 59] ODC is also linked to tumor progression. Therefore, a substantial reduction in ODC activity can inhibit tumor growth.[36] The products of the methionine salvage pathway negatively regulate ODC. The inhibition of ODC by MTA can be partially mediated by its metabolite MTOB from the MSP in yeast and tumor cells.[35, 46, 56]

Although not well investigated in human cancer cell lines and tissues, the metal dependence of ARD is clearly important in regulating MSP function.[6, 7, 18] The on-MSP form of ARD sustains the high polyamine synthesis rate essential for cell proliferation and provides methionine for SAM and protein production in normal cells. High polyamine synthesis rates lead to higher MTA concentrations, in turn requiring upregulation of MTAP to maintain proper MTA levels. Furthermore, functional MTA production suggests proper ODC regulation for tumor suppression.

A recent study has shown that acireductone-generating enzyme ENOPH1 (enolase-phosphatase 1) is also associated with cell cycle regulation. Knockdown of ENOPH1 suppressed cell proliferation and migration in malignant glioma and promoted ADI1GP translocation from the nucleus to cytoplasm, leading to an indirect decrease in MT1-MMP activity. [60] Zhang et. al. analyzed cultured brain microvascular endothelial cells in rat under oxygen-glucose deprivation (OGD) induced ENOPH1 upregulation. However, knockdown ENOPH1 had no effect on OGD-induced *ADI1* upregulation. On the other hand, ENOPH1 was found to regulate OGD-induced ADI1GP translocation from the nucleus to the cytoplasm. ENOPH1 knockdown increased the permeability of the endothelial monolayer to ADI1GP. It was hypothesized that reduced ADI1GP concentrations in the cytoplasm would dysregulate MT1-MMP located on the cell membrane.[61]

## 4. Carbon monoxide as an anti-apoptotic signal: A potential off-MSP role for ADI1GP in carcinogenesis

With the exception of the potentiation of HCV infection, nearly all of the reports summarized above suggest that ADI1GP plays a protective role in carcinogenesis and tumor metastasis, or at least enables other directly protective processes to occur. However, if metals other than Fe(II) are bound in the active site of *Hs*ARD (e.g., Ni(II), Mn(II), Co(II)), the off-MSP products, 3-(methylthio)propionate and carbon monoxide (CO), are the dominant products of acireductone dioxygenation.[5, 6] In this regard, CO is of particular interest. While toxic at high levels, CO is now generally accepted to be a cellular gasotransmitter at low concentrations, modulating inflammatory and apoptotic responses to cell damage.[62-64] In particular, CO is thought to prevent mitochondrial membrane permeabilization, the first step in cytochrome c-initiated apoptosis.[62]

This raises the intriguing possibility that off-MSP production of CO by Ni- or Mn-bound *Hs*ARD might be cytoprotective for carcinomas, while also rationalizing the methionine dependence of many cancer cell lines. In this regard, the Ni-bound ARD is of particular interest. The Ni-bound *Hs*ARD is the most thermostable of all metal-bound forms of the enzyme, with a midpoint for denaturation curves at 61 °C, with the Fe-bound form at 52 °C.[6] Furthermore, removal of Fe(II) from the ARD active site occurs under mild conditions, while Ni can only be removed by denaturation.[3] As such, nickel binding to ADI1GP would be both a thermodynamic and kinetic sink, permanently preventing expressed *Hs*ARD from binding iron and catalyzing the on-MSP chemistry required for methionine salvage. While Mn^2+^-bound HsARD is less thermostable than the Fe-bound enzyme (denaturation midpoint at 46 °C), the relative abundance of manganese relative to nickel in the cell means it may also represent a reasonable pathway for off-pathway CO production. To date, the only confirmed source of endogenous CO is heme oxygenase-1 (HO-1), which oxidatively breaks down hemin to CO and bilirubin, and endogenous CO production via an off-MSP route has not been detected in normal mammalian tissues. We are currently testing this possibility in transformed tissues, particularly those from Met-dependent cell lines.

Another consideration arises in light of the significant conformational differences that are observed between Fe- and Ni-bound bacterial ARD (and inferred from the differential chromatographic behavior and thermal stabilities of the corresponding *Hs*ARDs).[4, 6] As virtually all of the experimental data obtained thus far regarding the role(s) of ADI1GP in carcinogenesis, apoptosis and metastasis have been obtained in cellular cultures, it is not known whether any of the roles of ADI1GP discussed here are dependent upon which metal is present in the active site (or indeed if any metal is present at all). A case can be plausibly made that, for example, regulatory binding of ADI1GP to MMP-MT1 could be modulated by the bound metal, particularly if the conformational differences between metal ARD isozymes involves the MMP-MT1 binding site, which is still unknown.

## 5. Conclusions

Although *ADI1* gene expression and ADI1GP in multiple type of cells and tissues have been shown to correlate (often negatively) with cancer migration, metastasis and apoptosis, it is not clear whether these effects are due directly to ADI1GP interactions with other cellular components, or indirectly, as a result of enzymatic function, either on- or off-MSP. SAM, MTA, ODC, L-methionine, MTOB, polyamines, ENOPH1 and ADI1GP have all been implicated in regulation of processes such as cell division, migration, metastasis and apoptosis. It is also possible that it is a combination of factors that link ADI1GP to cancer.

Probably the most important unknown is the role of metal binding to ADI1GP in any of these observations. None of the cell or tissue studies have identified what, if any, metal is bound to ADI1GP. The prostate cancer study [22] indicated that while mutation of the four metal ligands in ADI1GP destroys enzymatic activity, it does not affect pro-apoptotic and colony inhibition. Both the HCC and PDAC studies [24, 42] showed the ADI1GP binds to the cytoplasmic tail of MT1-MMP, suggesting that this function, at least, is independent of metal binding. However, for the bacterial enzyme, at least, the Fe-bound and apo-ARDs forms are isostructural [4] leaving open the question of whether Ni or Mn binding could interfere with the ADI1GP-MT1-MMP interaction.

The following questions remain to be investigated:

1. Does the metal-dependent on/off MSP divergence occur in human cell lines? If so, under what conditions? Is off-MSP chemistry observed only under certain circumstances (e.g., in transformed cells)?
2. Is the methionine dependence of many cancer cell lines the result of off-MSP chemistry?
3. Do different metals bound to the ADI1GP act as switches for regulation of cell processes such as division, migration, invasion, metastasis and apoptosis? Is it possible to distinguish normal cells from tumor cells based on the metal content of their ADI1GP?
4. Does binding of metals other than Fe^2+^ change the interaction between ADI1 and MT1-MMP?

A systems biology approach will be necessary to place all of these possibilities into a complex cellular context, but answering some of these questions will help to define the role(s) of ADI1GP and, particularly, *Hs*ARD in human health.

## Figures and Tables

**Table 1.** Observed methionine dependence of various cancer cell lines.

Citation	Type of cancer	Cell lines	Met dependence (Ccomplete, ++, partial. +independent, −
Najim et al. (2009)[8]	Central nervous system	Daoy (medulloblastoma)	++
		D-54 (glioma)	++
Kokkinakis et al. (2001)[9]	Brain	D-54	++
		SWB77 (glioblastoma)	++
		Daoy	++
Willmann et al.(2015)[10]	Breast cancer	epithelial cell line MCF-10A	++
Kano et al. (1982)[11]	Leukemia	Raji (Burkitt)	++
		BALL (B-cell)	++
		TALL (T-cell)	++
		MOLT-3 (T-cell) (−)	−
		MOLT 4B (T-cell)	++
		HL60 (promyelocytic )	++
		K562 (chronic myelogenous leukemia in blastic crisis)	++
Poirson-Bichat et al.(2000)[12]	Colon, lung, glioma	TC71-MA (colon)	++
		SCLC6 (small cell lung)	++
		SNB19 (glioma)	++
*Lu et al.(2000)*[13]	Prostate	LNCaP (lymph node metastasis)	−
		PC-3 (distant metastasis)	++
		DU 145(distant metastasis)	+
*Guo et al.(1993)*[14]	Prostate, lung, fibrosarcoma	PC3	++
		SKLU-I (lung carcinoma)	++
		HT 1080 (fibrosarcoma)	++
*Mecham et al.(1983)*[15]	Bladder, breast, cervical, colon, kidney, lung, prostate, fibrosarcoma, osteogenic sarcoma, glioblastoma, neuroblastoma	EJ (bladder)	++
		J82 (bladder)	++
		SK-BR-2-II (breast)	++
		MCF-7 (breast)	++
		HeLa (cervical)	++
		SK-CO-1 (colon)	++
		A498 (kidney)	++
		A2182 (lung)	++
		PC-3	++
		8387 (fibrosarcoma)	++
		HT-1080	++
		HOS (osteogenic sarcoma)	++
		Human neurological tumors	++
		A172 (glioblastoma)	++
		SK-N-SH (neuroblastoma)	++

**Table 2.** *ADI1* and ADI1GP dependences of cancer cell lines.

Types of Cancer	Tissue/ Cell type	ADI1GP expression	Metastatic/ apoptotic	Mechanism
Hepatocellular carcinoma	Tissue , J7 and Huh7	Down regulated by CAV1, overexpressed	No information	Not proposed
Prostate cancer[22, 31]	Tissue, LNCaP and PC3	Observed in epithelial cells, little/no expression in stromal cells. Expression may be Mib regulated, Gleason gr. 3 prostate tumor tissue < ADI1GP than benign tissue.	Apoptotic	Associated with metal binding
Pancreatic ductal adenocarcinoma[42]	DanG, BxPC3, and Panc-1	Overexpressed	Metastatic	ADI1GP MT1-MMP interaction
